# Nanoparticles Using Lavender and Rosemary Extracts as Reducing Agent: Physicochemical Characterization and Antiproliferative Activity Against Human Cancer Cells

**DOI:** 10.3390/nano16211391

**Published:** 2026-10-29

**Authors:** Lívia Mačák, Jana Ondrašovičová, Martina Zigova, Marek Vojtko, Maksym Lisnichuk, Silvia Ondrašovičová, Peter Pal’ove-Balang, Ján Király

**Affiliations:** 1 Department of Biomedical Engineering and Measurement, Faculty of Mechanical Engineering, Technical University of Košice, Letná 9, 042 00 Košice, Slovakia; 2 Department of Stomatology and Maxillofacial Surgery, Faculty of Medicine, Pavol Jozef Šafárik University in Košice, 041 90 Košice, Slovakia; jana.ondrasovicova@upjs.sk; 3 Department of Pharmacology, Faculty of Medicine, Pavol Jozef Šafárik University in Košice, Trieda SNP 1, 040 11 Košice, Slovakia; martina.chripkova@upjs.sk; 4 Division of Ceramic and Non-Metallic Systems, Institute of Materials Research, Slovak Academy of Sciences, Watsonova 47, 040 01 Košice, Slovakia; mvojtko@saske.sk; 5 Institute of Physics, Faculty of Science, Pavol Jozef Šafárik University in Košice, Park Angelinum 9, 040 01 Košice, Slovakia; maksym.lisnichuk@upjs.sk or mlisnichuk@saske.sk; 6 Institute of Materials Research, Slovak Academy of Sciences, Watsonova 47, 040 01 Košice, Slovakia; 7 Department of Biology and Physiology, University of Veterinary Medicine and Pharmacy in Košice, 041 81 Košice, Slovakia; 8 Institute of Biology and Ecology, Faculty of Science, Pavol Jozef Šafárik University in Košice, 041 54 Košice, Slovakia; peter.palove-balang@upjs.sk; 9 Department of Microbiology and Immunology, University of Veterinary Medicine and Pharmacy in Košice, 041 81 Košice, Slovakia; jan.kiraly@uvlf.sk

**Keywords:** silver nanoparticles, green synthesis, lavender, rosemary, anticancer activity, antiproliferative activity, cancer cells, MTT assay

## Abstract

Silver nanoparticles (AgNPs) synthesized using plant extracts represent a promising approach combining environmentally friendly nanoparticle synthesis with potential biomedical applications. In this study, AgNPs were synthesized using aqueous extracts of dried *Lavandula angustifolia* (LEx) and *Salvia rosmarinus* (REx) leaves, physicochemically characterized, and evaluated for their antiproliferative activity against six human cancer cell lines and one non-tumorigenic human cell line. HPLC analysis revealed distinct phytochemical profiles of the two extracts, while UV–Vis, FTIR, TEM, SAED, and SEM–EDX analyses were used to characterize the resulting lavender-mediated (LAg) and rosemary-mediated (RAg) AgNPs. TEM analysis showed a greater proportion of smaller nanoparticles in RAg than in LAg. Antiproliferative activity was evaluated using the MTT assay after 48 h of exposure, with LEx, REx, and AgNO_3_ included as controls. Both AgNP formulations exhibited cell-line-dependent activity. LAg reached IC_50_ values of 19.4–20.8 µmol Ag/L in five cancer cell lines, whereas RAg reached IC_50_ values of 14.4–25.2 µmol Ag/L. The lowest IC_50_ was observed for RAg in PaTu 8902 pancreatic adenocarcinoma cells (14.4 ± 0.6 µmol Ag/L). In contrast, the IC_50_ was not reached in A549 or non-tumorigenic CCD-18Co cells at concentrations up to 50 µmol Ag/L (IC_50_ > 50 µmol Ag/L). Neither LEx nor REx produced 50% inhibition of cell viability at concentrations up to 1.7% (*v*/*v*). Similarly, AgNO_3_ did not achieve 50% inhibition at concentrations up to 50 µmol/L in any of the cell lines tested. These findings demonstrate formulation- and cell-line-dependent antiproliferative activity of green-synthesized AgNPs and support further investigation of their biological effects and underlying mechanisms.

## 1. Introduction

Cancer remains a major global health challenge, and the development of therapeutic strategies capable of effectively targeting malignant cells while minimizing adverse effects on healthy tissues continues to be an important objective of biomedical research. Despite substantial advances in cancer treatment, conventional therapeutic approaches may be limited by systemic toxicity, insufficient tumour selectivity, and the development of treatment resistance. Consequently, nanotechnology-based approaches have attracted considerable attention as potential tools for the development of novel anticancer strategies [1,2,3,4].

Among metallic nanomaterials, silver nanoparticles (AgNPs) have gained increasing interest because of their distinctive physicochemical and biological properties. In addition to their well-established antimicrobial activity, AgNPs have demonstrated cytotoxic and antiproliferative effects against various cancer cell types [4,5,6,7,8]. Their biological effects are strongly influenced by physicochemical characteristics, including particle size, morphology, surface charge, aggregation state, surface chemistry, and Ag^+^ ion release. These parameters can affect cellular uptake, intracellular distribution, and interactions with cellular components, thereby influencing cellular responses to AgNP exposure [9,10].

The anticancer effects of AgNPs have been associated with several interconnected cellular mechanisms. One of the most frequently reported mechanisms involves the generation of reactive oxygen species (ROS) and subsequent disruption of intracellular redox homeostasis. Excessive oxidative stress has been associated with mitochondrial dysfunction, membrane and DNA damage, alterations in cell-cycle progression, and activation of apoptotic pathways [11,12,13]. However, the magnitude of these effects can vary substantially depending on nanoparticle properties, exposure concentration and duration, and the biological characteristics of the investigated cell type. Therefore, the physicochemical characteristics of AgNPs should be considered together with their biological effects when evaluating their potential biomedical applications [14,15,16]. In parallel with investigations of their biological effects, increasing attention has been directed toward environmentally sustainable approaches for AgNP synthesis. Conventional chemical synthesis may involve reducing and stabilizing agents that generate undesirable residues, whereas green synthesis using biological materials provides an alternative approach in which naturally occurring compounds participate in the reduction of Ag^+^ ions and stabilization of the resulting nanoparticles. Plant extracts are particularly attractive for this purpose because they contain diverse bioactive compounds, including phenolic compounds, flavonoids, terpenoids, and other redox-active molecules that may contribute to nanoparticle formation and surface stabilization [17,18,19,20]. The composition of the plant extract, together with reaction parameters such as pH, temperature, reaction time, and precursor concentration, can substantially influence nanoparticle size, morphology, stability, and surface characteristics [21,22]. *Lavandula angustifolia* (lavender) *and Salvia rosmarinus* (rosemary) are rich in biologically active phytochemicals and therefore represent suitable plant sources for the green synthesis of AgNPs. Differences in phytochemical composition between plant extracts may influence nanoparticle nucleation, growth, surface functionalization, and size distribution and may consequently affect their interactions with biological systems [23,24]. Comparing AgNPs synthesized using different plant sources under controlled synthesis and biological testing conditions may therefore provide useful information on formulation-dependent differences in their physicochemical properties and biological effects.

Green-synthesized AgNPs have increasingly been investigated for their potential anticancer activity. Recent experimental studies have demonstrated antiproliferative effects of plant-mediated AgNPs against different human cancer cell models [25], while broader studies indicate that their effects may involve oxidative stress, mitochondrial impairment, DNA damage, cell-cycle alterations, and apoptosis [26]. Nevertheless, cellular responses depend strongly on both the nanoparticle formulation and the investigated cell type. Direct comparison of AgNPs synthesized using different plant extracts under identical experimental conditions may therefore help identify formulation-dependent differences in antiproliferative activity. Furthermore, evaluation across multiple cancer cell lines together with a non-tumorigenic cell line can provide preliminary information on differences in cellular sensitivity [27,28].

Therefore, the present study aimed to synthesize and physicochemically characterize AgNPs using aqueous extracts of *Lavandula angustifolia* and *Salvia rosmarinus* and to comparatively evaluate their antiproliferative activity against six human cancer cell lines representing melanoma, breast, pancreatic, colorectal, and lung cancers. The corresponding plant extracts and AgNO_3_ were included as controls. In addition, the non-tumorigenic CCD-18Co human fibroblast cell line was included to provide a preliminary comparison of the response of cancer and non-tumorigenic cells. The study further examined differences in sensitivity among the tested cell lines in relation to the physicochemical characteristics of the synthesized AgNPs.

## 2. Materials and Methods

### 2.1. Materials

Silver nitrate (AgNO_3_, purity > 98%; Mikrochem Ltd., Pezinok, Slovakia) was used as the silver precursor for nanoparticle synthesis. Dried leaves of *Lavandula angustifolia* (lavender) and *Salvia rosmarinus* (rosemary) were used for the preparation of aqueous plant extracts. The leaves were collected from plants grown on the campus of the Technical University of Košice (TUKE), Košice, Slovakia, where both plant species were cultivated at the same location under comparable environmental conditions. Deionized water was used for the preparation of the plant extracts and all aqueous solutions used during AgNP synthesis.

### 2.2. Preparation of Lavender and Rosemary Extracts

Dried leaves of *Lavandula angustifolia* and *Salvia rosmarinus* were used separately for the preparation of aqueous plant extracts. Prior to extraction, the plant material was washed twice with deionized water to remove surface impurities and subsequently mechanically crushed using a mortar. For each extract, 1.25 g of dried plant material was mixed with 50 mL of deionized water. The mixtures were heated at approximately 80 °C for 10 min under continuous stirring at 600 rpm. After cooling to room temperature, the extracts were filtered through filter paper to remove solid plant residues and subsequently centrifuged at 9000 rpm for 10 min. The resulting supernatants were collected. The *L. angustifolia* extract was designated as LEx, whereas the *S. rosmarinus* extract was designated as REx. The extracts were used for AgNP synthesis, and aliquots were retained for subsequent FTIR and HPLC analyses.

### 2.3. Green Synthesis of Silver Nanoparticles

AgNP colloids were prepared separately using LEx and REx. An aqueous precursor solution containing Ag^+^ at a concentration of 500 mg Ag/L was prepared from AgNO_3_ in deionized water and heated to approximately 80 °C under continuous stirring at 600 rpm.

After reaching the required temperature, LEx or REx was added to the silver precursor solution at a precursor-to-extract volume ratio of 10:1. The reaction mixtures were subsequently maintained at approximately 70 °C for 30 min under continuous stirring. Immediately after the reaction, the solutions were cooled to room temperature in a water bath. AgNP formation was accompanied by a visible color change from light yellow to dark brown. The resulting colloidal suspensions were allowed to cool to room temperature and were subsequently used for physicochemical characterization and biological testing. AgNPs synthesized using LEx were designated as LAg, whereas AgNPs synthesized using REx were designated as RAg. The resulting LAg and RAg colloidal suspensions were not subjected to post-synthesis washing or purification prior to biological testing. The synthesis of both LAg and RAg was independently repeated six times under the same experimental conditions.

### 2.4. Characterization of Silver Nanoparticles

Fourier-transform infrared spectroscopy (FTIR) was used to characterize the principal functional groups present in LEx and REx and to compare the spectra of the plant extracts with those of the corresponding LAg and RAg systems following AgNP synthesis. The infrared spectra were recorded using a Bruker Tensor 27 FTIR spectrometer (Bruker Optics GmbH, Ettlingen, Germany) equipped with a DTGS KBr detector. For each sample, 64 scans were collected in absorbance mode over the spectral range of 4000–400 cm^−1^ at a resolution of 4 cm^−1^. Samples were prepared using the KBr pressed-disc technique. Approximately 0.1 mg of each sample was dispersed in 150 mg of KBr and pressed into 13 mm diameter pellets. The spectra were evaluated comparatively with respect to changes in band positions and relative intensities following synthesis.

Nanoparticle morphology and particle size were examined by transmission electron microscopy (TEM; JEOL JEM-2000FX, Tokyo, Japan) operated at 200 kV. Particle size distributions were determined from independent TEM micrographs for each sample using ImageJ software (version 1.54g, National Institutes of Health, Bethesda, MD, USA). Selected-area electron diffraction (SAED) was used to further examine the crystalline structure of the synthesized AgNPs. Particle-size distributions were determined from 20 TEM images acquired from randomly selected fields of view for each sample. A total of 2951 individual AgNPs for LAg and 2569 individual AgNPs for RAg were measured using ImageJ software.

Surface morphology and local elemental composition were further examined by scanning electron microscopy (SEM) using a JSM-IT810 scanning electron microscope (JEOL Ltd., Tokyo, Japan) equipped with an energy-dispersive X-ray spectroscopy (EDX) system. For SEM–EDX analysis, the AgNP suspensions were deposited dropwise onto aluminum substrates, dried, and subsequently carbon-coated. SEM imaging was performed using a secondary electron detector (SED) at an accelerating voltage of 20 kV and a working distance of 4.0 mm. Representative high-magnification images were acquired at up to 200,000× magnification. Local EDX analyses were performed using the EDX system of the SEM at an accelerating voltage of 20 kV and a working distance of 10.0 mm. The Al and C signals detected in the EDX spectra originated predominantly from the aluminum substrate and the carbon coating, respectively.

### 2.5. HPLC Analysis of Plant Extracts

Aqueous extracts were filtered through a 0.2 µm nylon membrane and subsequently analyzed by reversed-phase high-performance liquid chromatography (RP-HPLC) using an Agilent 1260 Infinity Quaternary LC system equipped with a 1260 Infinity diode-array detector (DAD) (Agilent Technologies, Santa Clara, CA, USA). Separation was performed on a Kromasil 100-5-C18 column (250 × 4.6 mm, 5 µm particle size Nouryon, Bohus, Sweden). The mobile phases consisted of 5% acetonitrile with 0.1% trifluoroacetic acid (A) and 100% acetonitrile with 0.1% trifluoroacetic acid (B), delivered at a flow rate of 0.8 mL/min. The column temperature was maintained at 30 °C. The gradient elution program was as follows: 0 min (90:10, A:B); 2 min (84:16); 10 min (78:22); 32 min (70:30); 45 min (0:100); 58 min (0:100); and 60 min (90:10).

Compounds were identified based on their retention times and UV absorption spectra and, where authentic standards were available, by comparison of their retention times with those of authentic external standards. The standards employed were gallic acid, 4-hydroxybenzoic acid, chlorogenic acid, neochlorogenic acid, cryptochlorogenic acid, p-coumaric acid, luteolin-7-O-glucuronide, eriodictyol, cirsimaritin, and carnosol (Sigma-Aldrich, St. Louis, MO, USA), as well as apigenin, apigenin-7-O-glucoside, luteolin, luteolin-7-O-glucoside, naringin, naringenin, caffeic acid, catechin, epicatechin, herniarin, and umbelliferone (Extrasynthese, Genay, France). Chromatograms were evaluated at 210, 280, 330, or 350 nm, depending on the specific absorption properties of the target compounds.

Quantification was performed using external calibration curves, and the results were expressed as molar concentrations (nmol/mL) in the extracts. For compounds lacking authentic standards, tentative identification was performed based on their characteristic UV spectra and chromatographic elution order, allowing their assignment to specific chemical classes. These compounds were classified and reported as derivatives of their respective parent structures. Their concentrations were estimated using the calibration curve of the structurally most similar available standard and expressed as equivalents (nmol/mL).

### 2.6. Cell Culture

Six human cancer cell lines and one non-tumorigenic human cell line were used in this study. HCT116 (CCL-247™, American Type Culture Collection (ATCC), Manassas, VA, USA) human colorectal carcinoma cells were cultured in RPMI 1640 medium (Biosera, Kansas City, MO, USA). A549 (CCL-185™) human alveolar adenocarcinoma, MDA-MB-231 (HTB-26™) human triple-negative breast adenocarcinoma, A2058 (ECACC 91100402, European Collection of Authenticated Cell Cultures, Salisbury, UK) human malignant melanoma, Caco-2 (ATCC HTB-37™) human colorectal adenocarcinoma, and CCD-18Co (CRL-1459™) non-tumorigenic human intestinal fibroblasts were cultured in high-glucose Dulbecco’s Modified Eagle Medium (DMEM) supplemented with sodium pyruvate (Biosera, Kansas City, MO, USA). PaTu 8902 human pancreatic adenocarcinoma cells, kindly provided by Prof. K. Smetana (Institute of Anatomy, Charles University, Prague, Czech Republic), were cultured in high-glucose DMEM supplemented with sodium pyruvate and 25 mM HEPES (Sigma, Steinheim, Germany).

All culture media were supplemented with 10% fetal bovine serum (FBS; Gibco, Thermo Fisher Scientific, Rockford, IL, USA) and antibiotic–antimycotic solution (Merck, Darmstadt, Germany). Cells were maintained at 37 °C in a humidified atmosphere containing 5% CO_2_.

### 2.7. MTT Assay

The effects of LAg and RAg on cellular metabolic viability were evaluated using the MTT assay. LEx, REx, and AgNO_3_ were included as controls. Cells were seeded into 96-well polystyrene plates (SARSTEDT, Nümbrecht, Germany) at a density of 5 × 10^3^ cells per well and allowed to adhere for 24 h.

For LAg and RAg, treatment concentrations were expressed as nominal silver-equivalent concentrations (µmol Ag/L). These concentrations were calculated from the initial Ag^+^ concentration of the AgNO_3_ precursor solution (500 mg Ag/L, corresponding to approximately 4.64 mmol Ag/L) and the precursor-to-extract volume ratio of 10:1 used during synthesis. After addition of the plant extract, the resulting LAg and RAg colloidal suspensions had a calculated nominal silver concentration of approximately 4.21 mmol Ag/L. The colloidal suspensions were subsequently diluted in culture medium to obtain nominal silver-equivalent treatment concentrations of 1, 5, 10, 20, 25, and 50 µmol Ag/L. These values represent the nominal silver content of the colloidal suspensions and do not represent the molar or number concentration of individual AgNPs.

LEx and REx were tested as corresponding extract controls using the same dilution scheme as that applied to the respective LAg and RAg treatments. Because the aqueous plant extracts are complex phytochemical mixtures without a defined molecular weight, their concentrations were expressed as volume fractions (% *v*/*v*) of the original aqueous extracts in the final culture medium. Based on the applied dilution scheme, the highest tested concentration of both LEx and REx was 1.7% *v*/*v*. AgNO_3_ was included as an ionic silver control and was tested at final concentrations of 1, 5, 10, 20, 25, and 50 µmol/L under the same culture and exposure conditions.

Cells were exposed to the respective treatments for 48 h. Following treatment, 10 µL of MTT solution (5 mg/mL; Sigma-Aldrich Chemie, Steinheim, Germany) was added to each well, and the plates were incubated for 4 h at 37 °C. Subsequently, 100 µL of 10% sodium dodecyl sulfate (SDS) solution was added to each well, followed by an additional 12 h incubation. Absorbance was measured at 540 nm using a Cytation™ 3 Cell Imaging Multi-Mode Reader (BioTek, Winooski, VT, USA). All experiments were performed independently three times (*n* = 3 biological replicates), with each concentration tested in triplicate wells (technical replicates). Technical replicates were averaged within each experiment and normalized to the corresponding untreated control. Data are expressed as mean ± SD of three independent experiments. IC_50_ values were determined by nonlinear regression using a four-parameter logistic (4PL) model.

## 3. Results and Discussion

### 3.1. Phytochemical Profile of Plant Extracts by HPLC

RP-HPLC-DAD analysis revealed distinct phytochemical profiles for LEx and REx. A total of 21 compounds or derivatives were quantified in LEx, whereas 16 compounds or derivatives were quantified in REx. The two extracts differed considerably in both the composition and relative abundance of their phytochemical constituents. The major compounds with a relative abundance exceeding 5% are summarized in Table 1.

LEx exhibited a diverse phytochemical profile. The predominant component was a hydroxycinnamic acid derivative detected at a retention time (RT) of 7.6 min, reaching 771.9 nmol/mL and accounting for 16.3% of the total quantified compounds. Other major constituents included a coumarin derivative at RT 12.9 min (678.4 nmol/mL; 14.3%), cryptochlorogenic acid at RT 8.8 min (613.6 nmol/mL; 13.0%), and another coumarin derivative at RT 25.7 min (587.7 nmol/mL; 12.4%). Rosmarinic acid-related compounds were also abundant, including a rosmarinic acid derivative at RT 12.4 min (514.2 nmol/mL; 10.8%) and rosmarinic acid at RT 20.1 min (503.4 nmol/mL; 10.6%). A further coumarin derivative detected at RT 10.4 min was present at a concentration of 255.8 nmol/mL and accounted for 5.4% of the total quantified compounds. The presence of hydroxycinnamic acids, rosmarinic acid, and related phenolic constituents is consistent with previously reported phytochemical profiles of *L. angustifolia* [29,30]. In contrast, REx was characterized by the predominance of rosmarinic acid, detected at RT 20.3 min at a concentration of 157.2 nmol/mL and representing 38.83% of the total quantified compounds. Caffeic acid was the second most abundant constituent (46.7 nmol/mL; 11.54%), followed by naringenin (33.6 nmol/mL; 8.30%). Two chromatographically resolved eriodictyol glycosides, detected at RT 16.4 and 17.0 min, accounted for 6.94% and 5.95%, respectively. The predominance of rosmarinic acid together with the presence of caffeic acid and other phenolic constituents is in agreement with the characteristic phytochemical composition reported for *S. rosmarinus* and related Lamiaceae extracts [31,32,33].

**Figure 1:**
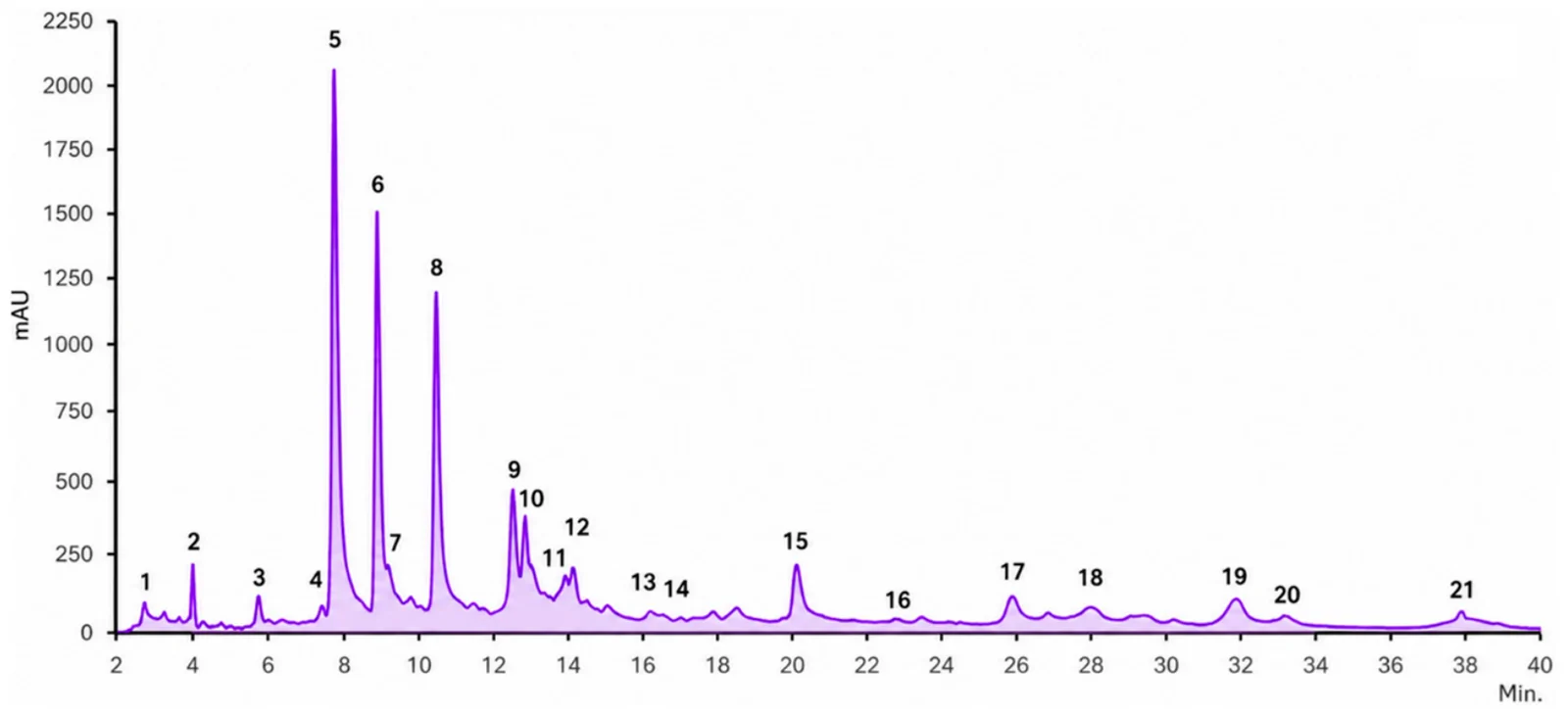
RP-HPLC-DAD chromatographic profile of LEx recorded at 280 nm (1, 4-hydroxybenzoic acid; 2, gallic acid; 3, neochlorogenic acid; 4, chlorogenic acid; 5, hydroxycinnamic acid derivative; 6, cryptochlorogenic acid; 7, caffeic acid; 8, coumarin derivative; 9, rosmarinic acid derivative; 10, coumarin derivative; 11, luteolin-7-O-glucoside; 12, 6-hydroxyluteolin-7-O-glycoside; 13, umbelliferone; 14, naringenin 7-O-glucoside; 15, rosmarinic acid; 16, apigenin glycoside; 17, coumarin derivative; 18, naringenin derivative; 19, naringenin derivative; 20, herniarin; 21, naringenin).

**Figure 2:**
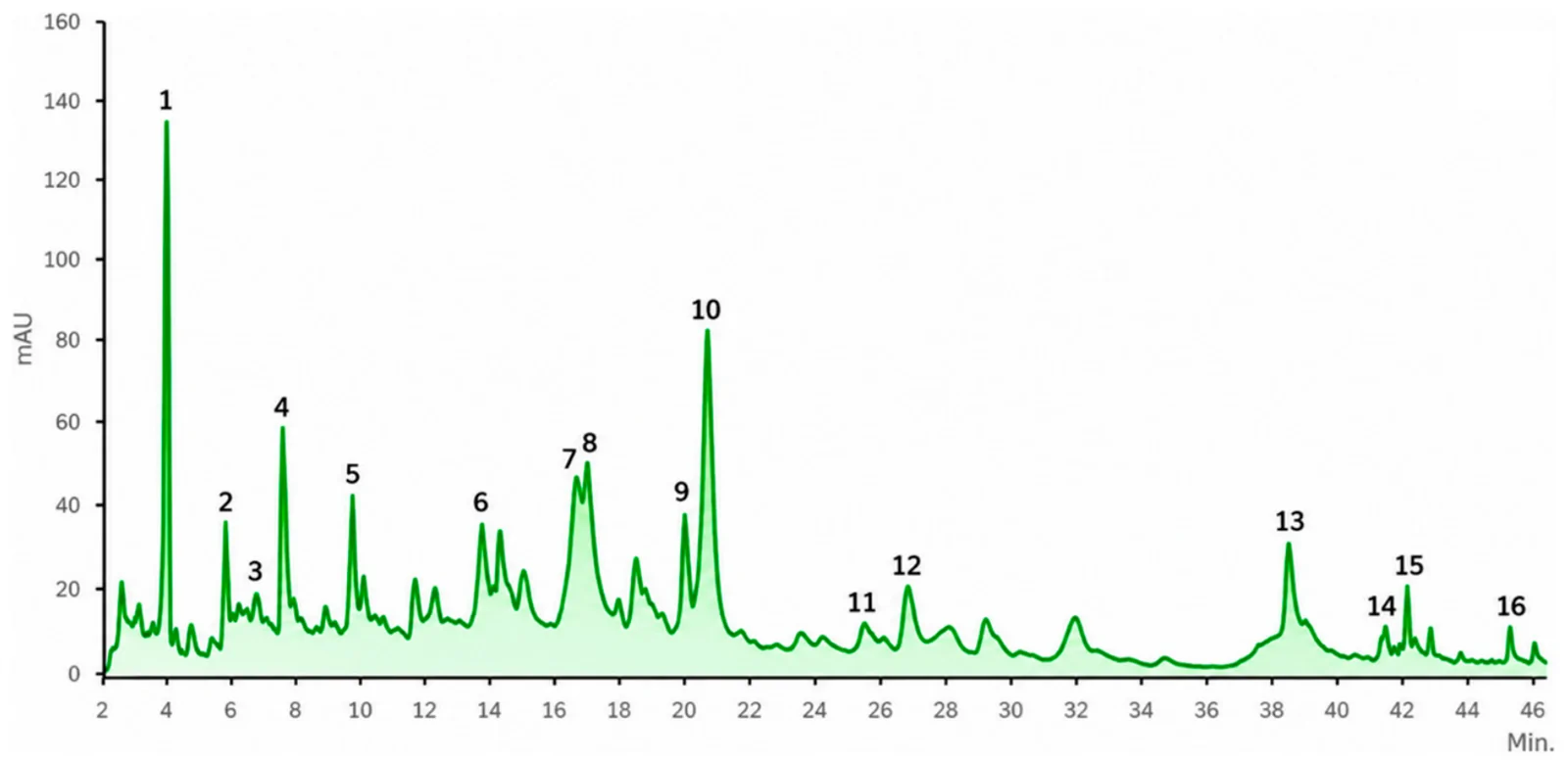
RP-HPLC-DAD chromatographic profile of REx recorded at 280 nm (1, gallic acid; 2, catechin; 3, neochlorogenic acid; 4, coumaric acid derivative; 5, caffeic acid; 6, luteolin-7-O-glucuronide; 7, eriodictyol glycoside; 8, eriodictyol glycoside; 9, apigenin derivative; 10, rosmarinic acid; 11, apigenin derivative; 12, apigenin derivative; 13, naringenin; 14, cirsimaritin; 15, carnosol or derivative; 16, lipophilic caffeic acid derivative).

The RP-HPLC-DAD chromatographic profiles recorded at 280 nm further demonstrated differences in the composition and distribution of phytochemical constituents between LEx and REx (Figure 1 and Figure 2).

### 3.2. FTIR Analysis

FTIR analysis revealed differences between the spectra of LEx and REx and those of the corresponding AgNP formulations, LAg and RAg (Table 2). LEx exhibited a broad absorption band at 3370 cm^−1^, which shifted to 3400 cm^−1^ in LAg. The band at 1420 cm^−1^ shifted to 1390 cm^−1^, whereas minor shifts were observed from 1260 to 1263 cm^−1^ and from 1050 to 1052 cm^−1^. The bands at 2930, 1560, 580, and 530 cm^−1^ remained at approximately the same positions. REx exhibited absorption bands at 3354, 2927, 1720, 1603, 1526, 1401, 1264, 1118, and 817 cm^−1^. Following AgNP synthesis, the band at 1720 cm^−1^ shifted to 1709 cm^−1^ in RAg, while the band at 1526 cm^−1^ shifted to 1520 cm^−1^. A band at 1075 cm^−1^ was observed in RAg, whereas the bands at 1401 and 817 cm^−1^ detected in REx were not clearly distinguishable after synthesis. The bands at 3354, 2927, 1603, 1264, and 1118 cm^−1^ remained at approximately unchanged positions. The observed spectral changes occurred mainly in regions tentatively associated with O–H, C=O, aromatic, and C–O-containing functional groups, which are commonly reported in FTIR analyses of plant-mediated AgNPs [22,25,26]. These changes indicate alterations in the chemical environment of plant-derived compounds following AgNP synthesis. However, because the LAg and RAg colloidal suspensions were not purified after synthesis, the detected spectral features may originate from both AgNP-associated and residual plant-derived components. Therefore, FTIR analysis does not allow individual compounds or their specific roles in AgNP formation and stabilization to be conclusively identified and cannot be considered direct evidence of a defined organic coating on the AgNP surface.

Assignments are tentative —indicates that a corresponding band was not clearly distinguishable in the respective spectrum.

### 3.3. Green Synthesis and UV–Vis Characterization

**Figure 3:**
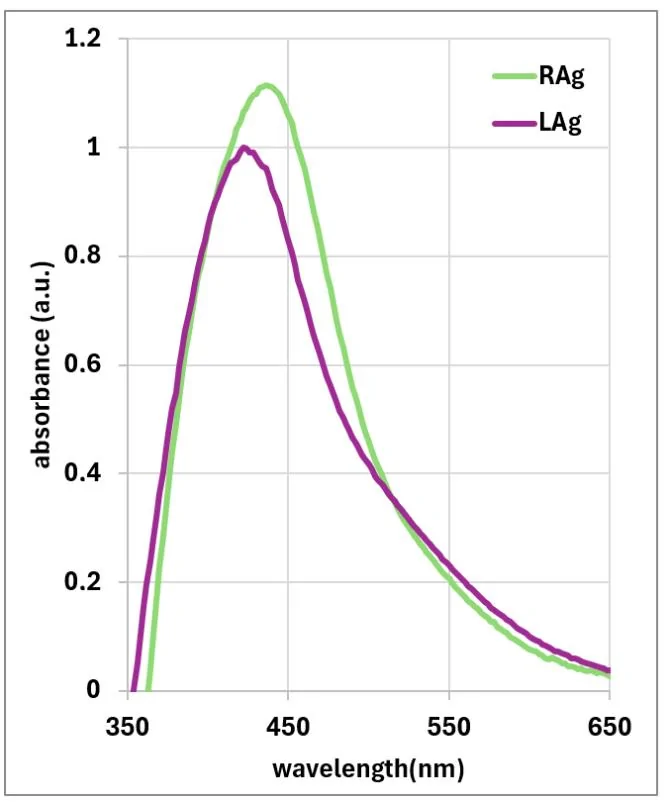
UV–Vis absorption spectra of LAg and RAg, showing SPR absorption maxima (λmax) at 426 and 442 nm, respectively.

The formation of AgNPs was initially indicated by a visible color change in the reaction mixtures from light yellow to dark brown during synthesis. Such color changes during plant-mediated synthesis of AgNPs are commonly associated with the development of surface plasmon resonance (SPR) as AgNPs are formed [34,35]. UV–Vis spectroscopy was subsequently used to evaluate the optical properties of LAg and RAg. Both AgNP formulations exhibited characteristic SPR absorption bands in the visible region (Figure 3), consistent with those commonly reported for plant-mediated AgNPs [25].

LAg exhibited an SPR absorption maximum at 426 nm, whereas RAg showed a maximum at 442 nm. The presence of these absorption bands was consistent with the formation of AgNPs using both LEx and REx. The difference in the SPR maximum between LAg and RAg indicates differences in their optical response. Since the position and shape of the SPR band of AgNPs can be influenced by several factors, including particle size, morphology, aggregation state, and the surrounding chemical environment [33,36,37], the observed spectral difference should not be attributed to particle size alone. The physicochemical differences between LAg and RAg were therefore further evaluated by TEM.

### 3.4. TEM and SAED Analysis

**Figure 4:**
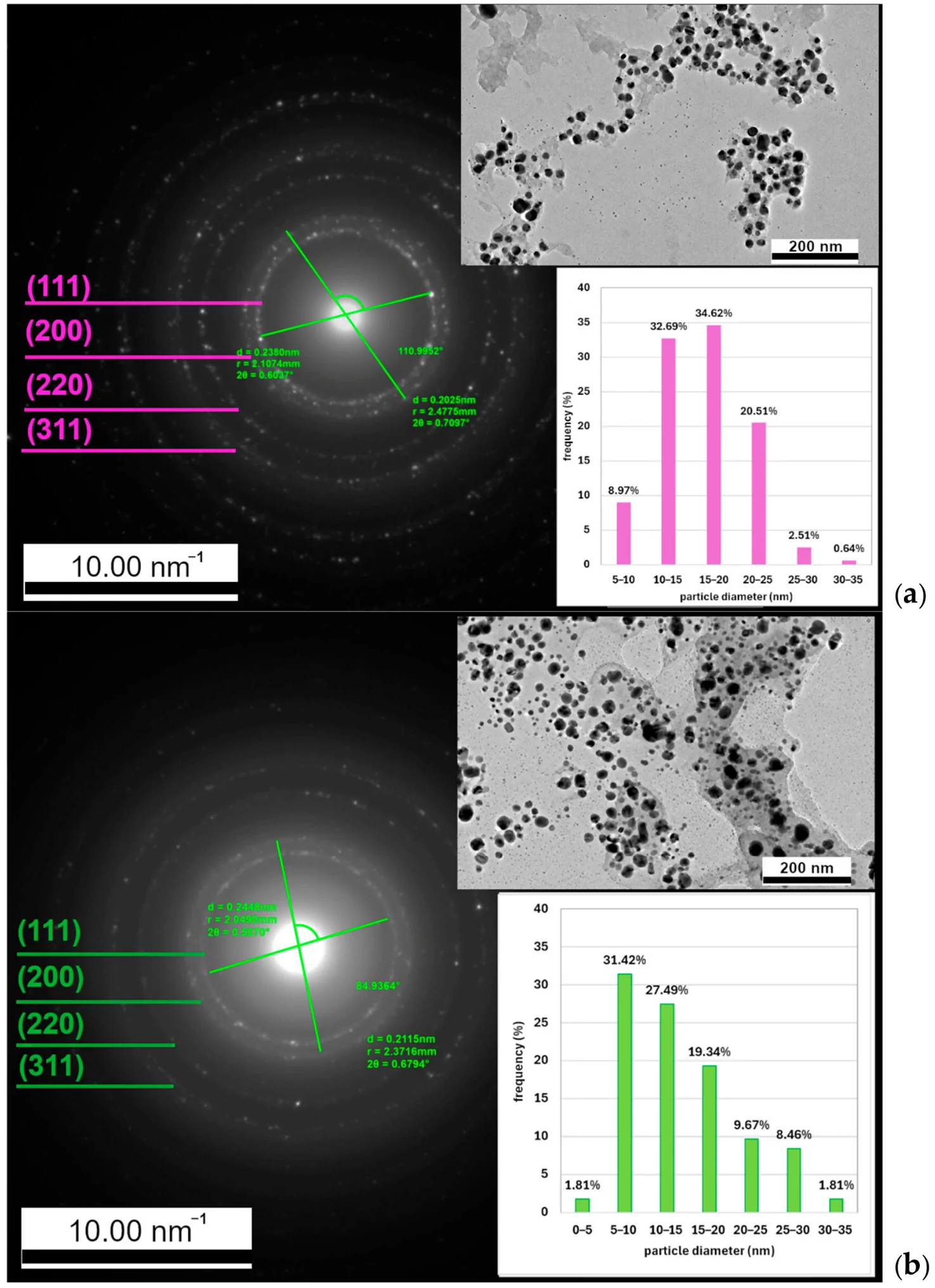
TEM and SAED characterization and particle-size distributions of LAg (**a**) and RAg (**b**).

TEM analysis revealed that both LAg and RAg consisted predominantly of spherical to quasi-spherical nanoparticles, with differences in their particle size distributions (Figure 4). The nanoparticles were observed both individually and in locally clustered regions.

LAg exhibited particle sizes ranging from 5.47 to 30.90 nm, with a mean particle diameter of 16.16 ± 4.81 nm. The size distribution was predominantly concentrated within the 10–25 nm range, which accounted for 87.82% of the analyzed nanoparticles. The highest relative frequency was observed in the 15–20 nm interval (34.62%), followed by the 10–15 nm (32.69%) and 20–25 nm (20.51%) intervals.

RAg exhibited a broader particle size range of 4.16–49.03 nm, with a mean particle diameter of 14.59 ± 7.40 nm. The distribution was shifted toward smaller particle sizes compared with LAg. The highest relative frequency occurred in the 5–10 nm interval (30.82%), followed by the 10–15 nm (27.49%) and 15–20 nm (19.34%) intervals. Overall, 58.31% of the analyzed RAg nanoparticles were within the 5–15 nm range. Thus, despite its broader overall size range, RAg exhibited a lower mean particle diameter and a greater proportion of smaller nanoparticles than LAg.

SAED analysis further supported the crystalline character of both AgNP formulations (Figure 4). The diffraction patterns exhibited multiple concentric rings composed of discrete diffraction spots, consistent with the polycrystalline character of the analyzed nanoparticle populations. For LAg, interplanar spacings of 0.2380 and 0.2025 nm were measured and assigned to the (111) and (200) planes, respectively, by comparison with the reference diffraction data for fcc metallic Ag (JCPDS/PDF No. 04-0783). For RAg, the measured interplanar spacings of 0.2448 and 0.2115 nm were consistent with the (111) and (200) planes, respectively.

Overall, TEM and SAED analyses demonstrated that both LAg and RAg consisted predominantly of nanoscale, crystalline particles, while revealing differences in their particle size distributions. In particular, RAg contained a greater proportion of smaller nanoparticles, whereas LAg exhibited a narrower overall particle size range.

### 3.5. SEM–EDX Analysis

**Figure 5:**
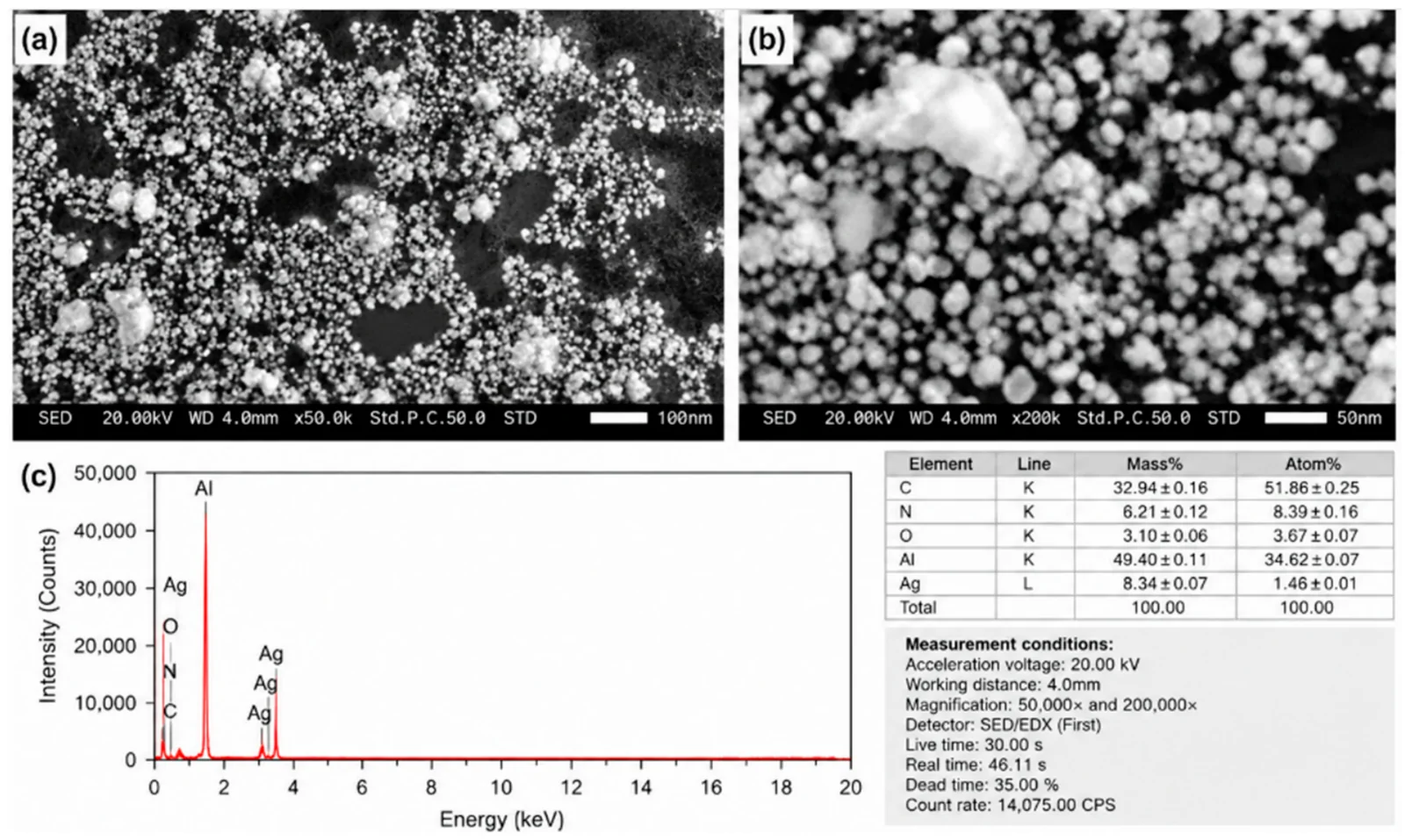
SEM–EDX characterization of LAg and RAg. (**a**) SEM micrograph of LAg, (**b**) SEM micrograph of RAg, and (**c**) EDX spectrum and elemental composition of LAg.

SEM analysis was performed to further examine the morphology of the green-synthesized AgNPs. Representative SEM micrographs acquired at different magnifications are shown in Figure 5. At 50,000× magnification, the sample exhibited densely distributed nanoscale features forming larger clustered regions. Higher-magnification imaging at 200,000× provided a more detailed view of these structures and showed closely associated nanoscale particles. The SEM observations were consistent with the tendency of the nanoparticles to form aggregates during sample preparation and drying.

EDX analysis performed on a selected region confirmed the presence of Ag (Figure 5). The analyzed region contained 8.34 ± 0.07 wt.% Ag, corresponding to 1.46 ± 0.01 at.%. Signals of C, N, O, and Al were also detected. The strong Al contribution (49.40 ± 0.11 wt.%) originated predominantly from the aluminum substrate, while the C signal (32.94 ± 0.16 wt.%) may also have been influenced by the carbon coating used during sample preparation. Because the LAg suspension was not purified after synthesis, the detected C, N, and O signals cannot be specifically attributed to organic compounds bound to the AgNPs and may also originate from residual plant-derived components. Since EDX was performed locally, the measured elemental composition represents only the selected analysis region and should not be interpreted as the bulk composition of the AgNP sample.

### 3.6. Effects of LAg and RAg on Cellular Metabolic Viability

The effects of LAg and RAg on cellular metabolic viability were evaluated after 48 h of exposure in six human cancer cell lines (A2058, MDA-MB-231, PaTu 8902, Caco-2, A549, and HCT116) and one non-tumorigenic human cell line (CCD-18Co). LEx, REx, and AgNO_3_ were included as controls. The half-maximal inhibitory concentration (IC_50_) values are summarized in Table 3.

Both LAg and RAg produced cell-line-dependent reductions in metabolic cell viability. LAg reached measurable IC_50_ values in five of the six investigated cancer cell lines. IC_50_ values of 19.6 ± 1.2, 19.4 ± 0.9, 20.2 ± 1.7, 20.6 ± 1.2, and 20.8 ± 1.6 µmol Ag/L were determined for A2058, MDA-MB-231, PaTu 8902, Caco-2, and HCT116 cells, respectively. The IC_50_ for A549 cells was not reached within the investigated concentration range of up to 50 µmol Ag/L.

RAg also reached measurable IC_50_ values in five of the six investigated cancer cell lines. IC_50_ values of 19.6 ± 0.8, 19.6 ± 1.5, 14.4 ± 0.6, 25.2 ± 2.1, and 20.4 ± 1.4 µmol Ag/L were determined for A2058, MDA-MB-231, PaTu 8902, Caco-2, and HCT116 cells, respectively. The lowest IC_50_ value was observed for PaTu 8902 pancreatic adenocarcinoma cells (14.4 ± 0.6 µmol Ag/L). As observed for LAg, the IC_50_ for A549 cells was not reached within the investigated concentration range of up to 50 µmol Ag/L.

Neither LAg nor RAg reached an IC_50_ in the non-tumorigenic CCD-18Co cell line within the investigated concentration range of up to 50 µmol Ag/L. Similarly, LEx and REx did not reach an IC_50_ in any of the investigated cell lines within the tested concentration range of up to 1.7% *v*/*v*. AgNO_3_ also did not reach an IC_50_ in any of the investigated cell lines at concentrations up to 50 µmol/L under the experimental conditions used.

Overall, the MTT results demonstrate a cell-line-dependent response to LAg and RAg. Both formulations reached measurable IC_50_ values in five of the six investigated cancer cell lines, whereas the IC_50_ was not reached in A549 cancer cells or in non-tumorigenic CCD-18Co cells within the investigated concentration range.

## 4. Discussion

### 4.1. Characterization of LAg and RAg

HPLC analysis demonstrated marked differences in the phytochemical composition of LEx and REx used for AgNP synthesis. LEx exhibited a comparatively diverse phytochemical profile, with hydroxycinnamic acid and coumarin derivatives among the predominant constituents, together with cryptochlorogenic acid, rosmarinic acid, and a rosmarinic acid derivative. In contrast, REx was characterized by a high relative abundance of rosmarinic acid, which accounted for 38.83% of the total quantified compounds, followed by caffeic acid, naringenin, and two chromatographically resolved eriodictyol glycosides.

The presence of phenolic compounds and flavonoids is particularly relevant to plant-mediated AgNP synthesis. Plant extracts contain multiple classes of biomolecules that may participate in the reduction of Ag^+^ and stabilization of the resulting AgNPs. Their contribution depends on their chemical structure and concentration as well as the experimental conditions employed during synthesis. The phytochemical profiles observed in the present study therefore provide a chemically plausible basis for the ability of both LEx and REx to mediate AgNP formation. However, because the complete aqueous extracts rather than isolated compounds were used, the contribution of individual phytochemicals to Ag^+^ reduction and nanoparticle stabilization cannot be established from the present data.

The substantial compositional differences between LEx and REx may also have contributed to the physicochemical differences observed between LAg and RAg. In addition to extract composition, parameters such as pH, temperature, AgNO_3_ concentration, reaction time, and extract-to-precursor ratio can influence AgNPs nucleation, growth, morphology, and stabilization. Because LAg and RAg were synthesized under comparable experimental conditions in the present study, differences in extract composition represent one plausible factor contributing to their distinct physicochemical characteristics. Nevertheless, establishing a direct relationship between individual phytochemicals and AgNPs properties would require controlled experiments using isolated compounds or chemically defined mixtures.

Comparative FTIR analysis revealed changes in the position and relative intensity of several bands between LEx and LAg and between REx and RAg, indicating changes in the chemical environment of plant-derived functional groups during the synthesis process. However, because the colloidal suspensions were not purified after synthesis, these spectral differences cannot distinguish surface-associated organic compounds from residual extract components. The FTIR results therefore support changes in the organic component of the reaction system during AgNP synthesis but do not provide direct evidence of a defined phytochemical coating on the AgNP surface.

The formation of LAg and RAg was supported by UV–Vis spectroscopy, which revealed characteristic SPR absorption bands with maxima at 426 nm for LAg and 442 nm for RAg. SPR absorption in this spectral region is commonly associated with AgNP formation, while the exact position and shape of the absorption band can be influenced by particle size, morphology, aggregation state, and the surrounding chemical environment. Consequently, the difference between the SPR maxima of LAg and RAg should not be attributed solely to differences in particle size.

TEM analysis demonstrated predominantly spherical to quasi-spherical nanoparticles in both formulations but revealed distinct particle-size distributions. LAg exhibited particle sizes ranging from 5.47 to 30.90 nm, with a mean particle diameter of 16.16 ± 4.81 nm. Most LAg particles were distributed within the 10–25 nm range (87.82%), with the highest relative frequency in the 15–20 nm interval (34.62%). RAg exhibited a broader overall size range of 4.16–49.03 nm and a lower mean particle diameter of 14.59 ± 7.40 nm. Its distribution contained a greater proportion of smaller nanoparticles, with 58.31% occurring within the 5–15 nm range and the highest relative frequency in the 5–10 nm interval (30.82%). Thus, although RAg displayed a broader overall size range, its distribution was shifted toward smaller particle sizes compared with LAg. Similar predominantly spherical morphologies and nanoscale particle dimensions have been reported for AgNPs synthesized using different biological and plant-mediated approaches [37,38,39,40].

These differences between the optical and TEM characteristics of LAg and RAg illustrate the complexity of interpreting SPR position solely in terms of particle diameter. In green-synthesized AgNPs, the optical response may additionally be affected by particle morphology, polydispersity, aggregation, and the local dielectric environment created by plant-derived compounds associated with the nanoparticle system. The observed UV–Vis and TEM differences should therefore be considered complementary rather than interpreted through a simple direct relationship between SPR wavelength and mean particle size.

FTIR analysis provided additional evidence of changes in the chemical environment of plant-derived compounds following AgNP synthesis. In LAg, changes were observed particularly in spectral regions associated with O–H and C–O-containing functional groups, including a shift in the broad band from 3370 to 3400 cm^−1^ and a shift from 1420 to 1390 cm^−1^. For RAg, changes included shifts from 1720 to 1709 cm^−1^ and from 1526 to 1520 cm^−1^, together with the appearance of a band at 1075 cm^−1^. These spectral changes are consistent with alterations in the chemical environment of hydroxyl-, carbonyl-, aromatic-, and C–O-containing functional groups during plant-mediated AgNP synthesis.

Taken together with the HPLC results, the FTIR spectra are consistent with the participation of plant-derived organic compounds in the synthesis process. However, the observed spectral changes cannot identify which individual phytochemicals were directly involved in Ag^+^ reduction or determine which compounds remained associated with the AgNPs surface after synthesis. Such conclusions would require additional compound-specific or surface-sensitive analytical techniques.

SAED analysis further supported the crystalline character of both AgNP formulations. The diffraction patterns exhibited multiple concentric rings composed of discrete diffraction spots, consistent with the polycrystalline nature of the analyzed nanoparticle populations. For LAg, interplanar spacings of approximately 0.2380 and 0.2025 nm were obtained from selected diffraction rings, whereas for RAg, the corresponding values were approximately 0.2448 and 0.2115 nm. Similar SAED patterns and interplanar spacings have been reported for crystalline AgNPs synthesized using biological and plant-mediated approaches [37,38,39,40]. Overall, the observed diffraction features are consistent with the crystalline structure of metallic Ag.

SEM observations provided complementary information on the morphology of the dried AgNP samples. Both LAg and RAg showed densely distributed nanoscale features forming clustered regions, consistent with aggregation occurring during deposition and drying for SEM analysis. The higher-magnification RAg micrograph provided a more detailed visualization of the closely associated nanoscale structures. These SEM observations should be considered complementary to TEM rather than used for quantitative particle-size determination.

EDX confirmed the local presence of Ag in the analyzed LAg region. However, because the colloidal suspension was not purified after synthesis, the detected C, N, and O signals cannot be used as evidence of organic compounds bound to the AgNPs, as residual plant-derived components may also have contributed to these signals. Therefore, the EDX results were interpreted only as local elemental information and not as evidence of a defined organic coating on the AgNPs.

Overall, the combined HPLC, UV–Vis, FTIR, TEM, SAED, and SEM–EDX results demonstrate that LEx and REx produced AgNPs with distinct physicochemical characteristics. Particularly notable was the difference in particle-size distribution, with RAg exhibiting a greater proportion of smaller nanoparticles than LAg. These physicochemical differences may be relevant when interpreting the subsequent biological responses. Nevertheless, particle size alone is unlikely to account for differences in antiproliferative activity, as surface chemistry, Ag^+^ release, aggregation behavior under cell-culture conditions, and interactions with biological molecules may also influence cellular responses.

### 4.2. Effects of LAg and RAg: Comparison with Previous Studies

This study shows that silver nanoparticles synthesized from aqueous extracts of Lavandula angustifolia (LAg) and Salvia rosmarinus (RAg) exhibit pronounced, cell-line-dependent antiproliferative activity against a panel of human cancer cell lines. Both nanoparticle preparations reached measurable IC_50_ values in five of the six cancer cell lines investigated, whereas no IC_50_ was observed in the non-tumorigenic CCD-18Co cells at concentrations up to 50 µmol Ag/L. Importantly, the corresponding aqueous plant extracts (LEx and REx) and AgNO_3_ did not reach IC_50_ values in any investigated cell line within their respective tested concentration ranges. These findings indicate that the antiproliferative activity observed for LAg and RAg cannot be attributed solely to the plant extracts or to the silver precursor under the experimental conditions used, but appears to be associated with the formation of the phytogenic AgNPs.

Enhanced biological activity of plant-mediated AgNPs compared with the corresponding plant extracts has been reported in previous studies. Plant-derived phytochemicals, including phenolic compounds, flavonoids, terpenoids, proteins, and other secondary metabolites, may participate in Ag^+^ reduction and nanoparticle stabilization and may influence the surface chemistry of the resulting nanoparticle systems. Consequently, the resulting nanoparticle systems may exhibit surface-associated plant-derived components that, together with the silver nanostructure, may influence cellular uptake and biological activity [41,42,43]. A systematic review of plant-mediated metallic nanoparticles further demonstrated that their cytotoxic potency is strongly influenced by particle size, morphology, and the biological material used for synthesis. Thus, direct quantitative comparisons of IC_50_ values across studies should be interpreted cautiously because nanoparticle composition, size, surface chemistry, concentration units, exposure duration, and viability assays vary considerably among experimental systems [44].

The activity of LAg observed in the present study is consistent with previous findings demonstrating the cytotoxic effects of *L. angustifolia*-mediated AgNPs. Belova et al. synthesized AgNPs using aqueous extracts of *L. angustifolia* and reported cell line-dependent cytotoxicity in CHO, SK-BR-3 breast adenocarcinoma, SKOV3-1ip ovarian adenocarcinoma, and SKOV-kat cells. Interestingly, coating the nanoparticles with bovine serum albumin completely abolished their cytotoxic activity, indicating that nanoparticle surface accessibility and surface-mediated interactions play an important role in their biological effects. The authors also showed that the *L. angustifolia* extract and several of its chromatographic fractions exhibited cytotoxic activity and therefore proposed that plant-derived secondary metabolites associated with the nanoparticle surface may contribute to the observed effects [24]. In the present study, however, LEx alone did not reach an IC_50_ at concentrations up to 1.7% *v*/*v*, whereas LAg exhibited IC_50_ values of approximately 19–21 µmol Ag/L in A2058, MDA-MB-231, PaTu 8902, Caco-2, and HCT116 cells. These findings suggest that the antiproliferative activity of LAg cannot be explained by the crude lavender extract alone and may instead arise from physicochemical and biological properties acquired during nanoparticle formation, potentially including interactions between the silver nanostructure and plant-derived surface constituents [45].

Further support is provided by Simsek et al., who evaluated L. angustifolia-mediated AgNPs in U87MG human glioblastoma cells and reported a concentration-dependent reduction in cell viability, with an IC_50_ of 7.536 µg/mL after 72 h. In addition to growth inhibition, these nanoparticles increased p53 and activated caspases-3, -8, and -9, supporting apoptosis as an important mechanism underlying their cytotoxicity [46].

Recent data on R. officinalis-mediated AgNPs likewise support the activity observed by RAg. Elmehalawy et al. reported antiproliferative activity of rosemary-derived AgNPs against MDA-MB-231 and PANC-1 cancer cells, with IC_50_ values of 177.2 and 115.3 µg/mL, respectively. In contrast, non-tumorigenic Vero and Wi38 cells showed higher IC_50_ values (233 and 207 µg/mL, respectively) [47]. This pattern is particularly interesting in relation to the present results, because RAg was active against MDA-MB-231 cells (IC_50_ = 19.6 ± 1.5 µmol Ag/L) and exhibited its strongest effect in PaTu 8902 pancreatic adenocarcinoma cells (IC_50_ = 14.4 ± 0.6 µmol Ag/L). Thus, although the pancreatic cell models and nanoparticle preparations differ between the two studies, both indicate that pancreatic cancer cells are susceptible to rosemary-mediated AgNPs. The comparatively low IC_50_ of RAg in PaTu 8902 cells observed here warrants further investigation to determine whether this reflects enhanced nanoparticle uptake, differences in oxidative-stress handling, or another tumor-cell-specific vulnerability.

The response of MDA-MB-231 triple-negative breast cancer cells also aligns with the broader literature on this cell line’s susceptibility to AgNPs. Gurunathan et al. reported that biologically synthesized AgNPs reduced MDA-MB-231 cell viability in a concentration-dependent manner and induced ROS generation, LDH release, caspase-3 activation, nuclear fragmentation, and apoptosis [48]. The IC_50_ values of 19.4 ± 0.9 and 19.6 ± 1.5 µmol Ag/L for LAg and RAg, respectively, support MDA-MB-231 cells as a responsive model and indicate little difference between the two plant-mediated nanoparticle preparations in this cell line.

Both LAg and RAg also inhibited HCT116 colorectal carcinoma cells, with nearly identical IC_50_ values of 20.8 ± 1.6 and 20.4 ± 1.4 µmol Ag/L, respectively. HCT116 cells have repeatedly been shown to be sensitive to biogenic AgNPs. For example, Javed and Mashwani reported an IC_50_ of 1.7 µg/mL for Mentha arvensis-mediated AgNPs in HCT116 cells and demonstrated apoptosis and accumulation of cells in the sub-G1 phase [49]. Gurunathan et al. similarly showed that naringenin-mediated AgNPs reduced HCT116 viability by approximately 50% at 5 µg/mL and induced ROS generation, mitochondrial dysfunction, DNA damage, and alterations in p53-associated signaling [50].

Caco-2 cells were also responsive to both nanoparticle preparations, although RAg was less potent (IC_50_ = 25.2 ± 2.1 µmol Ag/L) than LAg (20.6 ± 1.2 µmol Ag/L). Previous studies have shown that AgNPs can reduce Caco-2 viability in a size-, concentration-, and exposure time-dependent manner [51,52]. The difference between LAg and RAg in Caco-2 cells, together with the opposite pattern in PaTu 8902 cells, where RAg was more active, further supports the conclusion that nanoparticle biological activity is cell line-dependent rather than reflecting a uniformly greater potency of one nanoparticle preparation.

An interesting finding was the comparatively low sensitivity of A549 lung adenocarcinoma cells. Neither LAg nor RAg reached an IC_50_ in A549 cells at concentrations up to 50 µmol Ag/L, in marked contrast to the five other cancer cell lines. This result does not imply that A549 cells are intrinsically resistant to AgNPs, as other AgNP preparations have demonstrated antiproliferative effects in this cell line. For example, Derris trifoliata-derived AgNPs showed moderate antiproliferative activity against A549 cells, with a reported median effective concentration of approximately 86.23 ± 0.22 µg/mL [53]. In contrast, Manilkara zapota-derived AgNPs inhibited A549 cells with an IC_50_ of approximately 29 µg/mL after 72 h [54]. The markedly different responses reported for A549 cells therefore emphasize the importance of nanoparticle physicochemical characteristics and experimental conditions. Differences in particle size, shape, surface charge, aggregation state, cellular uptake, dissolution and release of Ag^+^, and exposure duration can all modify the cellular response to AgNPs [41,42,43,44,45].

One of the most notable findings of the present study is the apparent differential response between malignant and non-tumorigenic cells. Neither LAg nor RAg reached an IC_50_ in CCD-18Co cells at concentrations up to 50 µmol Ag/L, whereas IC_50_ values between 14.4 and 25.2 µmol Ag/L were observed in five cancer cell lines. Similar preferential effects of phytogenic AgNPs on malignant cells have been reported in other experimental systems. For example, Manilkara zapota-derived AgNPs inhibited HCT116, HeLa, and A549 cancer cells without substantially affecting normal human lymphocytes and erythrocytes at the concentrations tested [54]. More recently, rosemary-derived AgNPs were reported to exhibit greater cytotoxicity toward breast and pancreatic cancer cells than toward normal Wi38 and Vero cells [47].

Nevertheless, the present results should be interpreted as evidence of an in vitro selectivity window rather than proof of tumor-specific cytotoxicity. Because the IC_50_ in CCD-18Co cells was not reached at the highest tested concentration, we cannot calculate an exact selectivity index. Based on the experimental range, the data indicate only a minimum selectivity ratio. For example, RAg showed an IC_50_ of 14.4 µmol Ag/L in PaTu 8902 cells, while the IC_50_ in CCD-18Co cells was >50 µmol Ag/L, corresponding to a minimum selectivity index of >3.47. For LAg, the lowest cancer-cell IC_50_ was 19.4 µmol Ag/L in MDA-MB-231 cells, corresponding to a minimum selectivity index of >2.58 relative to CCD-18Co cells. Confirming selective cytotoxicity will therefore require testing higher nanoparticle concentrations in CCD-18Co cells and, ideally, including additional non-tumorigenic cell types from tissues corresponding to the malignant models.

The absence of measurable IC_50_ values for AgNO_3_ within the investigated concentration range requires cautious interpretation. Although Ag^+^ ions are known to contribute substantially to the biological effects of silver-containing materials, their speciation and bioavailability can be strongly influenced by the composition of the cell culture medium. In chloride-containing media, Ag^+^ can interact with Cl^−^ to form poorly soluble AgCl and soluble silver–chloride complexes, thereby decreasing the fraction of freely available Ag^+^. In addition, serum proteins and other organic constituents of the culture medium may bind silver ions and further modify their bioavailability. Previous studies have demonstrated that silver cytotoxicity is strongly dependent on culture-medium composition and that chloride concentration and organic components can substantially influence the biological effects of ionic and nanosilver [41,42,43].

Therefore, the absence of a measurable IC_50_ for AgNO_3_ at concentrations up to 50 µmol/L in the present study may, at least partly, reflect reduced Ag^+^ bioavailability under the experimental conditions. However, silver speciation, AgCl formation, and the concentration of freely available Ag^+^ were not directly measured in this study; therefore, this explanation remains hypothetical. Moreover, components of the culture medium may also influence AgNP aggregation, stability, dissolution, and Ag^+^ release. Consequently, the greater reduction in metabolic cell viability observed for LAg and RAg compared with AgNO_3_ under the present experimental conditions should not be interpreted as direct evidence of intrinsically greater toxicity of the nanoparticle formulations. Further studies involving quantitative determination of dissolved and particulate silver under the exposure conditions are required to clarify these differences.

Mechanistically, numerous studies have associated the anticancer activity of AgNPs with increased intracellular ROS production, mitochondrial dysfunction, disruption of cellular antioxidant systems, DNA damage, cell-cycle perturbation, and activation of apoptotic pathways [42,43,48,49,50]. However, these mechanisms were not directly investigated in the present study. The MTT assay used here measures cellular metabolic activity and does not distinguish between inhibition of cell proliferation and induction of cell death. Therefore, the observed concentration-dependent reduction in metabolic cell viability cannot establish the molecular mechanisms responsible for the observed effects. The mechanisms described above should thus be regarded as hypotheses based on previous studies rather than conclusions from the present data. Future studies should include direct assessment of cellular Ag uptake, intracellular ROS generation, mitochondrial function, DNA damage, cell-cycle distribution, and appropriate cell-death markers to elucidate the mechanisms underlying the observed responses.

Overall, these findings show that AgNPs synthesized using *L. angustifolia* and *S. rosmarinus* extracts produced a substantially greater reduction in metabolic cell viability than the corresponding aqueous extracts or AgNO_3_ under the same experimental conditions. Both LAg and RAg reduced metabolic cell viability in five of the six cancer cell lines tested, whereas the IC_50_ was not reached in non-tumorigenic CCD-18Co cells within the tested concentration range. The particularly strong response of PaTu 8902 cells to RAg and the comparatively lower sensitivity of A549 cells further indicate that the biological response to phytogenic AgNPs depends on both nanoparticle characteristics and cellular context. Together with previous studies of lavender- and rosemary-mediated AgNPs, these results support further characterization of LAg and RAg, with particular emphasis on cellular Ag uptake, oxidative stress, apoptosis, and the physicochemical determinants underlying the observed differences in cellular response.

Although plant-mediated synthesis is commonly described as a green approach because plant-derived compounds can act as reducing and stabilizing agents and may reduce the need for conventional synthetic reagents, the use of plant extracts alone does not necessarily establish the overall environmental sustainability of the process. The environmental performance of such synthesis also depends on biomass availability and processing, extraction procedures, energy consumption, reaction conditions, waste generation, and scalability. In addition, seasonal and geographical variations in plant availability and composition may affect the standardization and reproducibility of plant-mediated nanoparticle synthesis. These factors represent important limitations when considering larger-scale production [55]. In the present study, no life-cycle assessment, energy balance, or direct environmental comparison with alternative synthesis routes was performed; therefore, the term “green synthesis” refers specifically to the plant-mediated synthesis approach and should not be interpreted as evidence of a quantitatively demonstrated environmental advantage over other synthesis methods.

HPLC analysis confirmed the presence of several potentially bioactive phytochemicals in the extracts, including hydroxycinnamic and coumarin derivatives, cryptochlorogenic and rosmarinic acids in LEx, and rosmarinic and caffeic acids, naringenin, and eriodictyol glycosides in REx. These compounds may participate in Ag^+^ reduction and stabilization of AgNPs and may potentially contribute to the biological properties of the resulting colloidal systems; however, their individual contributions were not investigated in the present study. The plant material was cultivated within the TUKE campus, collected during a single summer harvesting period, and a sufficient amount was dried and stored as one batch for use throughout the study, thereby reducing within-study variability related to geographical origin, growing conditions, and harvesting time. Nevertheless, geographical, seasonal, and batch-to-batch variations in phytochemical composition remain relevant for reproducibility and future scale-up, highlighting the importance of standardized plant material, extraction procedures, and phytochemical profiling.

A direct comparison with AgNPs synthesized using a defined reagent such as citric acid was not performed in the present study. Future studies should therefore compare plant-mediated and citrate-based AgNPs under matched synthesis and exposure conditions to determine their relative physicochemical properties, biological effects, reproducibility, and environmental performance.

### 4.3. Study Limitations and Future Perspectives

The present study provides a comparative evaluation of the physicochemical characteristics and in vitro antiproliferative activity of AgNPs synthesized using two phytochemically distinct plant extracts. Nevertheless, several limitations should be considered when interpreting the results.

First, antiproliferative activity was evaluated using the MTT assay as a single metabolic viability endpoint. Although this approach enabled quantitative comparison of IC_50_ values among the investigated treatments and cell lines, it does not distinguish between reduced metabolic activity, inhibition of cell proliferation, and cell death. Therefore, the observed decrease in MTT-derived cell viability should not be interpreted as evidence of a specific mechanism of action.

Second, the molecular mechanisms underlying the effects of LAg and RAg were not investigated. Consequently, the involvement of ROS generation, oxidative stress, mitochondrial dysfunction, DNA damage, cell-cycle alterations, apoptosis, or other pathways cannot be concluded from the present results. Although these mechanisms have been reported for AgNPs in other experimental systems, their involvement would require direct experimental verification for LAg and RAg. In addition, the LAg and RAg colloidal suspensions used for biological testing were not subjected to post-synthesis purification. Consequently, residual plant-derived components and silver species may have contributed to the observed effects on cellular metabolic viability, and the contribution of the AgNPs cannot be completely separated from that of the remaining reaction-medium components.

Third, only one non-tumorigenic cell line, CCD-18Co, was included in the present study. Although neither LAg nor RAg reached an IC_50_ in CCD-18Co cells within the investigated concentration range (>50 µmol Ag/L), this finding cannot be generalized to normal human cells. Evaluation of additional non-tumorigenic cell types will be necessary to determine whether the observed differential sensitivity extends beyond this individual cellular model. Another limitation is that the physicochemical behavior of LAg and RAg under cell-culture conditions was not directly characterized. Nanoparticle aggregation, interactions with serum proteins, protein-corona formation, and Ag^+^ release may alter the biological identity and cellular effects of AgNPs after their introduction into complete culture medium. Consequently, the physicochemical characteristics determined for the original AgNP formulations may not fully represent their state during cellular exposure. Future studies should therefore combine metabolic viability assays with complementary approaches, including apoptosis and necrosis analysis, intracellular ROS determination, mitochondrial membrane potential measurements, cell-cycle analysis, and appropriate molecular markers. Considering the lowest IC_50_ value observed for RAg in PaTu 8902 cells, further investigation of this AgNP–cell line combination would be particularly informative. Such studies could help determine whether the pronounced response of PaTu 8902 cells is associated with oxidative stress, mitochondrial dysfunction, apoptosis, or other cellular pathways. Further work should also evaluate the stability and transformation of LAg and RAg in complete cell-culture medium, including aggregation behavior, protein-corona formation, and Ag^+^ release. In addition, the inclusion of several non-tumorigenic cell lines representing different tissues would enable a more robust evaluation of differential cellular sensitivity. Finally, the present findings are limited to conventional in vitro cell-culture models. More complex experimental systems, including three-dimensional tumor models and, subsequently, appropriately designed in vivo studies, would be required before conclusions regarding therapeutic applicability, systemic safety, or anticancer efficacy could be drawn.

## 5. Conclusions

In this study, AgNPs were successfully synthesized using aqueous extracts of *Lavandula angustifolia* (LEx) and *Salvia rosmarinus* (REx), yielding two formulations, LAg and RAg, with distinct phytochemical and physicochemical characteristics. HPLC analysis revealed substantial differences between the extracts, with LEx exhibiting a diverse profile dominated by hydroxycinnamic acid and coumarin derivatives, whereas REx was characterized by a high relative abundance of rosmarinic acid. Complementary UV–Vis, FTIR, TEM, SAED, and SEM analyses confirmed the formation and provided information on the optical, surface-chemical, morphological, and crystalline characteristics of the resulting AgNPs, while local EDX analysis confirmed the presence of Ag in the analyzed region of LAg. TEM analysis revealed distinct particle-size distributions, with RAg exhibiting a lower mean particle diameter and a greater proportion of smaller nanoparticles than LAg.

Both LAg and RAg exhibited cell-line-dependent antiproliferative activity after 48 h of exposure. Measurable IC_50_ values were obtained in five of the six investigated cancer cell lines, whereas the IC_50_ was not reached in A549 cells within the investigated concentration range. The lowest IC_50_ value was observed for RAg in PaTu 8902 pancreatic adenocarcinoma cells (14.4 ± 0.6 µmol Ag/L). In contrast, neither LAg nor RAg reached an IC_50_ in the non-tumorigenic CCD-18Co cell line at concentrations up to 50 µmol Ag/L. Similarly, LEx, REx, and AgNO_3_ did not reach an IC_50_ in any of the investigated cell lines within the tested concentration range.

Overall, the findings demonstrate that the biological response to green-synthesized AgNPs depends on both the nanoparticle formulation and the cellular model. The comparatively strong response of PaTu 8902 cells to RAg, together with the absence of a measurable IC_50_ in CCD-18Co cells within the investigated concentration range, supports further investigation of this formulation. Future studies should address the underlying mechanisms of action, Ag^+^ release, nanoparticle behavior in biological media, and responses in additional non-tumorigenic and more complex cancer models before conclusions regarding selectivity, safety, or potential biomedical applicability can be established.

## Figures and Tables

**Table 1:** Major phytochemical constituents (>5% relative abundance) of LEx and REx determined by RP-HPLC-DAD.

Extract	Compound	Concentration (nmol/mL)	Relative Abundance (%)	RT (min)
*L. angustifolia*	Hydroxycinnamic acid derivative ^B^	771.9	16.3	7.6
	Coumarin derivative ^B^	678.4	14.3	12.9
	Cryptochlorogenic acid ^A^	613.6	13.0	8.8
	Coumarin derivative ^B^	587.7	12.4	25.7
	Rosmarinic acid derivative ^B^	514.2	10.8	12.4
	Rosmarinic acid ^A^	503.4	10.6	20.1
	Coumarin derivative ^B^	255.8	5.4	10.4
*S. rosmarinus*	Rosmarinic acid ^A^	157.2	38.83	20.3
	Caffeic acid ^A^	46.7	11.54	9.7
	Naringenin ^A^	33.6	8.3	38.3
	Eriodictyol glycoside I ^B^	28.1	6.94	16.4
	Eriodictyol glycoside II ^B^	24.1	5.95	17.0

Note: Relative abundance (%) was calculated as the percentage of the individual compound’s molar concentration relative to the total molar concentration of all quantified phenolic compounds. A Compound confirmed by comparison with an authentic external standard. B Compound tentatively identified based on its characteristic UV absorption spectrum and chromatographic elution order.

**Table 2:** FTIR absorption bands of LEx and REx and the corresponding green-synthesized AgNPs (LAg and RAg).

Sample	Extract (cm^−1^)	AgNPs (cm^−1^)	Tentative Assignment
LEx/LAg	3370	3400	O–H stretching
	2930	2930	Aliphatic C–H stretching
	1560	1560	Aromatic C=C/COO^−^ vibrations
	1420	1390	C–H bending/COO^−^ vibrations
	1260	1263	C–O stretching
	1050	1052	C–O/C–O–C stretching
	580	580	Fingerprint-region vibration
	530	530	Fingerprint-region vibration
REx/RAg	3354	3354	O–H stretching
	2927	2927	Aliphatic C–H stretching
	1720	1709	C=O stretching
	1603	1603	Aromatic C=C stretching
	1526	1520	Aromatic skeletal vibration
	1401	—	C–H bending/COO^−^ vibrations
	1264	1264	C–O stretching
	1118	1118	C–O stretching
	—	1075	C–O/C–O–C stretching
	817	—	Aromatic C–H out-of-plane bending

**Table 3:** Effects of silver nanoparticle formulations, corresponding plant extracts, and AgNO_3_ on the metabolic viability of human cancer and non-tumorigenic cells after 48 h of exposure.

Cell Line	LAg (µmol Ag/L)	LEx (% *v*/*v*)	RAg (µmol Ag/L)	REx (% *v*/*v*)	AgNO_3_ (µmol/L)
A2058	19.6 ± 1.2	>1.7	19.6 ± 0.8	>1.7	>50
MDA-MB-231	19.4 ± 0.9	>1.7	19.6 ± 1.5	>1.7	>50
PaTu 8902	20.2 ± 1.7	>1.7	14.4 ± 0.6	>1.7	>50
Caco-2	20.6 ± 1.2	>1.7	25.2 ± 2.1	>1.7	>50
A549	>50	>1.7	>50	>1.7	>50
HCT116	20.8 ± 1.6	>1.7	20.4 ± 1.4	>1.7	>50
CCD-18Co	>50	>1.7	>50	>1.7	>50

Values are expressed as mean IC_50_ ± SD from three independent experiments. The concentrations of LAg and RAg are expressed as nominal silver-equivalent concentrations (µmol Ag/L), calculated from the initial Ag^+^ concentration used during nanoparticle synthesis and the corresponding dilution factors. These values represent the nominal silver content of the colloidal suspensions and do not represent the molar or number concentration of individual nanoparticles. The concentrations of the aqueous plant extracts (LEx and REx) are expressed as volume fractions (% *v*/*v*) of the original extracts in the final culture medium. The extract controls were tested using dilution volumes corresponding to those used for the respective nanoparticle suspensions. Values > 50 µmol Ag/L for LAg and RAg, >50 µmol/L for AgNO_3_, or >1.7% *v*/*v* for LEx and REx indicate that the IC_50_ was not reached within the investigated concentration range. LAg, lavender-mediated silver nanoparticles; RAg, rosemary-mediated silver nanoparticles; LEx, aqueous *Lavandula angustifolia* extract; REx, aqueous *Salvia rosmarinus* extract.

## Data Availability

The original contributions presented in this study are included in the article. Further inquiries can be directed to the corresponding authors.

## Abbreviations

The following abbreviations are used in this manuscript:

AgNPs	Silver nanoparticles
LAg	Silver nanoparticles synthesized using lavender extract
RAg	Silver nanoparticles synthesized using rosemary extract
LEx	Lavender extract
REx	Rosemary extract
AgNO_3_	Silver nitrate
FTIR	Fourier-transform infrared spectroscopy
UV–Vis	Ultraviolet–visible spectroscopy
SPR	Surface plasmon resonance
TEM	Transmission electron microscopy
SEM	Scanning electron microscopy
EDX	Energy-dispersive X-ray spectroscopy
MTT	3-(4,5-Dimethylthiazol-2-yl)-2,5-diphenyltetrazolium bromide
IC_50_	Half-maximal inhibitory concentration
DMEM	Dulbecco’s Modified Eagle Medium
RPMI	Roswell Park Memorial Institute
FBS	Fetal bovine serum
HEPES	4-(2-Hydroxyethyl)-1-piperazineethanesulfonic acid
SDS	Sodium dodecyl sulfate
ROS	Reactive oxygen species
